# Chronic Kidney Disease as a Systemic Inflammatory Syndrome: Update on Mechanisms Involved and Potential Treatment

**DOI:** 10.3390/life11050419

**Published:** 2021-05-05

**Authors:** Francesca Tinti, Silvia Lai, Annalisa Noce, Silverio Rotondi, Giulia Marrone, Sandro Mazzaferro, Nicola Di Daniele, Anna Paola Mitterhofer

**Affiliations:** 1 Department of Translational and Precision Medicine, Sapienza University of Rome, Viale dell’Università 37, 00185 Rome, Italy; silvia.lai@uniroma1.it (S.L.); silveriorotondi@libero.it (S.R.); sandro.mazzaferro@uniroma1.it (S.M.); 2 Department of Systems Medicine, Nephrology and Dialysis Unit, University of Rome Tor Vergata, via Montpellier 1, 00133 Rome, Italy; annalisa.noce@uniroma2.it (A.N.); giulia.marrone@students.uniroma2.eu (G.M.); nicola.didaniele@uniroma2.it (N.D.D.); 3 PhD School of Applied Medical, Surgical Sciences, University of Rome Tor Vergata, via Montpellier 1, 00133 Rome, Italy

**Keywords:** chronic kidney disease, inflammation, acute kidney injury, cytokine, interleukin-6, mineral bone disease, fibroblast growth factor 23, oxidative stress, malnutrition, sarcopenia

## Abstract

Chronic kidney disease (CKD) is characterized by manifestations and symptoms involving systemic organs and apparatus, associated with elevated cardiovascular morbidity and mortality, bone disease, and other tissue involvement. Arterial hypertension (AH), diabetes mellitus (DM), and dyslipidemia, with glomerular or congenital diseases, are the traditional risk factors recognized as the main causes of progressive kidney dysfunction evolving into uremia. Acute kidney injury (AKI) has recently been considered an additional risk factor for the worsening of CKD or the development of CKD de novo. Evidence underlies the role of systemic inflammation as a linking factor between AKI and CKD, recognizing the role of inflammation in AKI evolution to CKD. Moreover, abnormal increases in oxidative stress (OS) and inflammatory status in CKD seem to exert an important pathogenetic role, with significant involvement in the clinical management of this condition. With our revision, we want to focus on and update the inflammatory mechanisms responsible for the pathologic conditions associated with CKD, with particular attention on the development of AKI and AKI-CKD de novo, the alteration of calcium-phosphorus metabolism with bone disease and CKD-MBD syndrome, the status of malnutrition and malnutrition–inflammation complex syndrome (MICS) and protein-energy wasting (PEW), uremic sarcopenia, the status of OS, and the different inflammatory pathways, highlighting a new approach to CKD. The depth comprehension of the mechanisms underlying the development of inflammation in CKD may present new possible therapeutic approaches in CKD and hopefully improve the management of correlated morbidities and provide a reduction in associated mortality.

## 1. Introduction

Chronic kidney disease (CKD) is a key contributor to morbidity and mortality in noncommunicable diseases, including diabetes mellitus (DM), metabolic syndrome, obesity, arterial hypertension (AH), and cardiovascular (CV) disease [1].

Kidney diseases exert significant consequences on global health, both as a direct cause of global morbidity and mortality and as an important risk factor for CV disease [2].

The prevalence of CKD has increased by about 30% since 1990 and is associated with rising treatment costs because of renal replacement therapy (RRT) and the long-term use of dialysis for patients with end-stage kidney disease (ESRD). The number of people receiving RRT exceeds 2.5 million and is projected to double to 5.4 million by 2030 [3].

Arterial hypertension, DM, and dyslipidemia, with glomerular or congenital diseases, are the traditional risk factors recognized as the main causes of progressive kidney dysfunction evolving into uremia [4].

Uremic CV risk factors are chronic causes of a low-grade inflammatory status associated with alteration in calcium-phosphorus metabolism, hyperhomocysteinemia, malnutrition, uremic sarcopenia, and oxidative stress (OS) [1].

Acute kidney injury (AKI) has recently been considered an additional risk factor for the worsening of CKD or the development of CKD de novo [5]. 

Evidence underlies the role of systemic inflammation as a linking factor between AKI and CKD, recognizing its role as responsible for AKI evolution to CKD. Moreover, abnormal increases in OS and inflammatory status in CKD seem to exert an important pathogenetic role, with significant involvement in the clinical management of this condition [6].

With our revision, we aim to focus on and update the dynamic complex of inflammatory mechanisms responsible for the pathologic conditions associated with CKD, with particular attention on the development of AKI and hypoxia, the alteration of calcium-phosphorus metabolism and bone disease, adipose tissue dysfunction, hyperhomocysteinemia, the status of malnutrition and uremic sarcopenia, gut microbiota alterations, and the status of OS. We consider the mutual relationship between CKD and the target organs as active effectors of kidney damage progression through systemic inflammatory mechanisms.

The depth comprehension of the mechanisms underlying the development of CKD may lead the way to specific treatments and hopefully to the improved management of correlated morbidities and reduction of associated mortality.

Alongside the recommended control of classical risk factors such as AH and hyperglycemia with common pharmacological tools, in particular angiotensin-converting enzyme (ACE) inhibitors or angiotensin-receptor blocker (ARB), we explore the potential role of the new renin–angiotensin system (RAAS) blocker mediated by the novel nonsteroidal receptor antagonist finerenone and sodium–glucose cotransporter-2 (SGLT-2) inhibition on CKD management.

**Figure 1:**
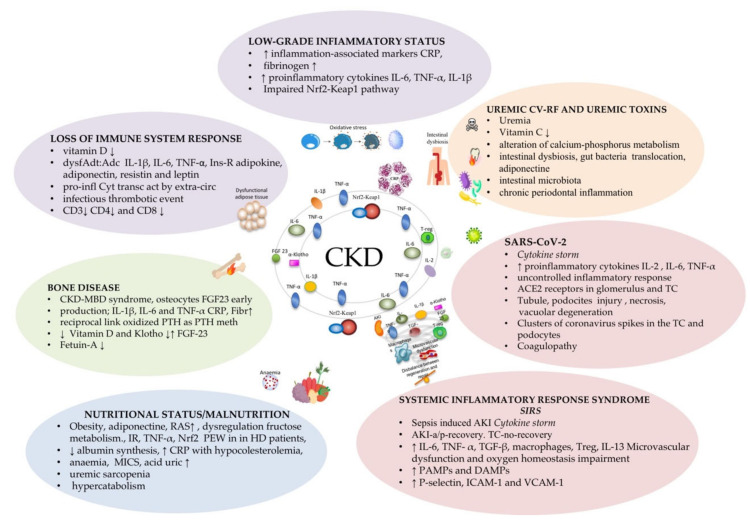
Chronic kidney disease as a systemic inflammatory syndrome. We describe the principal pathologic conditions connected to CKD and the related inflammatory mechanisms. a1-AG alpha1-acid glycoprotein; Adc: central adiposity; AKI: acute kidney injury; AKI-a/p-rec AKI apparent/partial recovery; CKD-MBD: chronic kidney disease-mineral bone disorder; CRP: C-reactive protein; Cyt: cytokines; DAMPs: damage-associated molecular patterns; dysf Adt: dysfunctional adipose tissue; E-Sel: E-selectin; FGF23: fibroblast growth factor 23; Fibr: fibrinogen; HD: hemodialysis; ICAM-1: intracellular adhesion molecule-1; IL: interleukin; Ins-res: insulin resistance; L-act: leukocyte activation; M: macrophages; MICS: malnutrition–inflammation complex syndrome; NGAL: neutrophil gelatinase-associated lipocalin; Nrf2-Keap1: Nrf2 nuclear factor erythroid 2 (NF-E2)-related factor 2 (Nrf2)–Keap1 (Kelch-like erythroid cell-derived protein with CNC homology (ECH)-associated protein) 1; OS: oxidative stress; PAMPs: pathogen-associated molecular patterns; perit-cap: peritubular capillaries; PEW: protein-energy wasting; PINI: prognostic inflammatory and nutritional index; Pres/Proc: presepsin/procalcitonin; P-sel: P-selectin; PTH: parathormone; PTH meth: PTH methionine-sulfone residues; RAS: TC: tubular cells; TGF: transforming growth factor; TNF: tumor necrosis factor; transc act: transcription activation; Treg: T-cell subsets regulatory T cells; VCAM-1: vascular cell adhesion molecule-1; ↑: increase; ↓: reduction.

The role of OS, hypoxia-induced factors (HIFs), and other inflammatory mediators are considered and described, as the kidney represents one of the most metabolically active organs, which renders it particularly vulnerable to oxidative damage, hypoxia, and inflammation. The main mechanisms underlying inflammation in CKD are summarized in Figure 1.

### 1.1. CKD and Systemic Inflammation

Increased morbidity and mortality associated with CKD are related to several factors, including traditional risk factors such as increased blood pressure, serum LDL cholesterol, body mass index, and the presence of cardiovascular disease, as well as newly recognized conditions, in particular malnutrition and chronic inflammation [7].

In CKD patients, systemic inflammation is characterized by a low-grade inflammatory status and seems to play a key role in increased morbidity and mortality associated with chronic nephropathy. Multiple factors can contribute to immune dysregulation and inflammatory activation in CKD. Alongside the role of traditional inflammatory biomarkers and the deregulated mineral metabolism characteristic, a role should be recognized for OS associated with CKD. In fact, the kidney is one of the most metabolically active organs, which renders it particularly vulnerable to oxidative damage, alteration of calcium-phosphorus metabolism, and bone disease, as well as hyperhomocysteinemia and malnutrition with uremic sarcopenia [8].

Chronic kidney disease is associated with the homeostatic deregulation of synthesis, release, and degradation of soluble molecules, including disturbances of the immune system with the disruption of cytokines and inflammatory mediators, and decreased renal clearance accounting for higher levels of circulating cytokines [9]. Interestingly, local proinflammatory cytokine production and an increased absorptive gradient have been demonstrated in the interstitial fluid of chronic peritoneal dialysis [10]. 

The progressive loss of kidney function during CKD demonstrates an inverse correlation with the increase in inflammation-associated markers, in particular C-reactive protein (CRP) and fibrinogen, and the increase in proinflammatory mediators, such as interleukin (IL)-6, tumor necrosis factor (TNF)-α, and IL-1β [11,12].

An association between albuminuria, kidney function, and inflammatory biomarkers has been found in CKD patients [13]. It has been shown that the increased release of inflammatory mediators is associated with the elevation of fibroblast growth factor (FGF)-23 levels. Increased levels of FGF-23, IL-6, and CRP independently predict mortality associated with CKD [14]. In fact, serum albumin, CRP, IL-6, and fetuin seem to be predictors of malnutrition, cardiovascular disease, and mortality in patients with advanced CKD [15].

Additional conditions associated with proinflammatory status are OS, produced by the uremic milieu, and metabolic acidosis, developing with a decrease in the glomerular filtration rate (GFR) [11]. A particular contributor is the emerging role of the impaired Nrf2 nuclear factor erythroid 2 (NF-E2)-related factor 2 (Nrf2)–Kelch-like erythroid cell-derived protein with CNC homology (ECH)-associated protein 1 (Keap1) pathway, which is one of the most important cell defense and survival pathways. Nrf2 can protect cells and tissues from a variety of toxicants by increasing the expression of a number of cytoprotective genes to OS and inflammation in CKD in relation to acidosis in hemodialysis (HD) patients [16,17]. Compounds that increase the activity of Nrf2 are being tested for disease prevention, whereas Nrf2 inhibitors are being developed as an adjuvant therapy to enhance the efficacy of, for example, chemotherapeutic drugs. Activation of the Nrf2 defense response has been shown to protect against neurodegenerative diseases, aging, DM, OS, CVD, and inflammation [18]. Accumulation of Nrf2 in cells creates an environment conducive for cell growth and protects against oxidative stress and toxic agents [19].

Uremic CV risk factors that exert a key role in CKD are associated with a chronic low-grade inflammatory status [20] responsible for the alteration of calcium-phosphorus metabolism, as observed in nondiabetic HD patients with coronary artery calcifications associated with long-term cardiovascular events [21,22], hyperhomocysteinemia [23], malnutrition [11,15], uremic sarcopenia [24], and OS [25,26]. 

Uremic toxins may also contribute to intestinal dysbiosis in CKD, facilitating the translocation of gut bacteria that can, in turn, activate the systemic inflammatory response [27]. During CKD, alterations in gut microbiota are associated with chronic periodontal inflammation, commonly developing in CKD patients, and are also associated with the systemic elevation of inflammatory biomarkers in HD patients and adversely affects patients’ survival [28].

Deficiency in vitamin D, which characterizes CKD, is proposed as an additional cause of altered inflammatory response as a result of the loss of the regulation of the immune system associated with vitamin D deficiency [29]. Vitamin D deficiency is a potential risk factor for noncommunicable diseases and viral infections [30]. It has been suggested that optimal serum levels of 25-hydroxyvitamin D have immunomodulatory and anti-inflammatory properties, and the possible benefit of vitamin D_3_ supplementation in coronavirus disease (COVID)-19 was explored [31].

A role for dysfunctional tissues in CKD, in particular the adipose tissue, is also responsible for the disrupted production of inflammatory cytokines. Patients with a GFR of 53.2 mL/min/1.73 m^2^ demonstrate overweight or obesity in 85% and DM in 40% of cases [32]. The relationship between obesity and dyslipidemia with CKD has been well-characterized. In particular, a relationship between subcutaneous adipose tissue, intrahepatic fat with inflammation, insulin resistance, and adipokine levels in CKD patients has been found [32].

In particular, obesity is equally associated with DM, AH, and abdominal obesity, and it is a known risk factor for CKD and progressive renal function loss. Central adiposity, especially in female patients, is considered one of the major drivers of cardiometabolic risk, characterized by increased markers of inflammation (IL-1β, IL-6, TNF-receptors 1 and 2), insulin resistance (homeostasis model assessment of insulin resistance), and increased adipokine levels (adiponectin, total and high molecular weight, resistin and leptin), all factors that contribute to inflammation with the impairment of renal function and mortality [33].

Nonalcoholic fatty liver disease (NAFLD) presents an additional model of systemic inflammation linking the liver and kidney, with a prevalence of CKD ranging between 25% and 35% [34].

Although NAFLD primarily affects patients with metabolic syndrome, obesity, and diabetes, recognizing classical risk factors such as obesity, renin–angiotensin system activation (RAS), fructose metabolism dysregulation, and lipogenesis in the development of both disorders, evidence considers inflammatory disorder the principal mechanism involved [34]. 

Patients with NAFLD and nonalcoholic steatohepatitis (NASH) present higher levels of TNF and TNF-messenger ribonucleic acid (mRNA) compared to healthy subjects. Impaired OS seems to play a key role in the pathogenesis of NAFLD and CKD, as well as the impairment of nuclear erythroid-related factor-2 (Nrf2) [35].

A correlation between insulin resistance and the necroinflammation and fibrosis of NASH is also described, characterized by increased IL-6, uric acid synthesis, increased adiponectin and adipose tissue inflammation with RAS activation, endothelial dysfunction, and tubulointerstitial fibrosis leading to CKD [36].

In a dialysis setting, HD treatment plays a particular role in the acute upregulation of proinflammatory cytokine transcription, both directly, by activation mediated by the extracorporeal circuit, and indirectly, mediated by the infectious and thrombotic events that frequently occur, creating additional inflammatory stimulation [37]. In these patients, several inflammatory biomarkers are associated with all-cause mortality, including, in particular, elevated levels of CRP and IL-6 and lower levels of fetuin-A and albumin [37,38,39,40]. 

### 1.2. CKD, Inflammation, and AKI

Contrary to previous studies, a causal link between AKI and the subsequent development or progression of CKD, recognizing that AKI and CKD are not distinct syndromes but two closely interconnected entities, was recently established. Observational studies have shown, independently of causes, that AKI leads to CKD de novo and the progression of pre-existing CKD, with increased mortality and associated CV disease [41,42,43].

Moreover, the severity of AKI predicts progression to CKD, and even small increases in the serum creatinine satisfying the criteria for AKI stage 1 enhance the risk of adverse short-term and long-term outcomes among patients with or without CKD [44,45].

**Figure 2:**
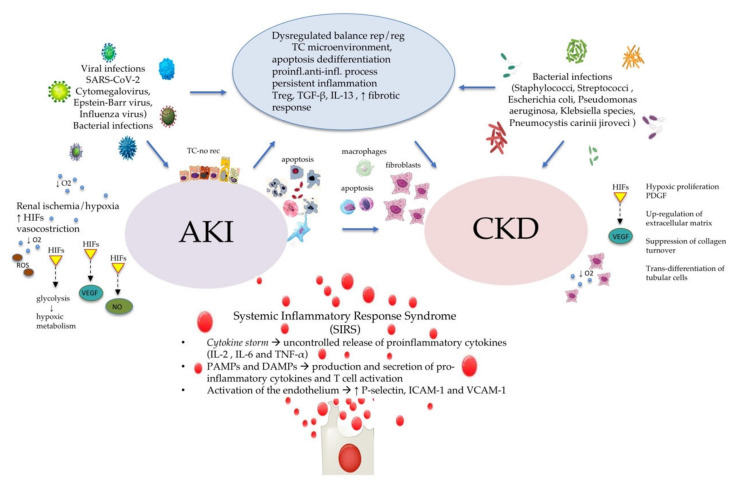
Acute to chronic kidney disease progression. Principal inflammatory mechanisms involved in acute to chronic kidney disease progression. AKI: acute kidney injury; AKI-a/p-rec AKI apparent/partial recovery; CKD: chronic kidney disease; DAMPs: damage-associated molecular patterns; HIFs: hypoxia-inducible factors; ICAM-1: intracellular adhesion molecule-1; IL: interleukin; O2: oxygen; OS: oxidative stress; NO: nitric oxide; PAMPs: pathogen-associated molecular patterns; VCAM-1: vascular cell adhesion molecule-1; ROS: reactive oxygen species; TGF-β: transforming growth factor-β; TNF-α: tumor necrosis factor-α Treg: T-cell subsets regulatory T cells; TC: tubular cell; VEGF: vascular endothelial growth factor; ↑: increase; ↓: reduction.

Mechanisms underlying this association are not completely understood, but studies in animals have highlighted a number of causal pathways. Experimental studies in animals have highlighted the role of tubular cells’ microenvironment as a result of the dysregulated balance between repair and regenerative pathways, apoptosis, dedifferentiation, and, in particular, proinflammatory and anti-inflammatory processes. Persistent inflammation with a high level of transforming growth factor (TGF)-β, macrophages, T-cell subsets, regulatory T cells (Treg), and IL-13 are the most important factors involved. The chronic dysregulation of these factors over time seems to determine a fibrotic response leading to CKD [46]. Principal mechanisms linking AKI to CKD are reported in Figure 2.

The role of systemic inflammation as a significant mechanism for the development of AKI has been described and defined over the last few years in systemic inflammatory response syndrome (SIRS) and sepsis, recognizing the central responsibility of sepsis-induced AKI in the setting of microvascular dysfunction with the release of microparticles, inflammation, and the energetic adaptation of highly metabolic organs to cellular stress [47]. 

AKI patients present raised levels of inflammatory mediators associated with poor prognosis, regardless of the cause of AKI, with IL-6 and TNF-α playing an important role in pathological changes occurring at the renal level [48]. Several blood purification devices, i.e., *Oxiris, Toraymyxin*, and *CytoSorb*, have demonstrated the capacity of removing both exogenous and endogenous inflammatory mediators, with improvement in clinical outcomes and in mortality risk [49].

A particular role in AKI and CKD is played by hypoxia [50,51]. Tissue hypoxia is a pathologic feature that can develop in several human tissues, including the kidney. Hypoxia-inducible factors (HIFs), in particular the subunits HIF-1α and 2α, are expressed in all tissues; HIF-2 is observed in tubular and collecting ducts, glomeruli, podocytes, and microvascular endothelial cells. HIFs are involved in the cellular response to hypoxia with the activation of numerous HIF genes responsible for metabolic and proliferative modification (hypoxic proliferation) through anaerobic glycolysis activation and vascular endothelial growth factor (VEGF) production. Erythropoietin was the first HIF gene identified, but many other hypoxia-inducible genes, such as VEGF, facilitated glucose transporter-1, adipose differentiation-related protein, adrenomedullin, and others have been described with direct relevance in adaptive or maladaptive process during AKI and CKD [52,53]. 

In ischemic injury linking AKI to different conditions such as sepsis or nephrotoxic and obstructive conditions, a depletion in ATP seems to correlate with tubular cell injury, the loss of brush border, and the unbounding of cell junctions caused by prolonged hypoxia, leading to the mitochondrial generation of reactive oxygen species (ROS) and resulting in cell dysfunction or death. Tissue-specific removal of HIF-1α in mice inhibits the development of tubulointerstitial disease, mesenchymal transition, and inflammation in response to ureteral obstruction [54].

In CKD, hypoxia seems to induce fibrosis by promoting upregulation of the extracellular matrix and through the suppression of collagen turnover and the transdifferentiation of tubular cells, which are features of CKD. Therefore, HIFs seem to play an important role in the progression of chronic renal disease [54].

The FGF-23 pathway may represent an additional link between the development of acute and chronic kidney disease. AKI, in fact, is associated with many of the mineral abnormalities observed in CKD such as hypocalcemia, hyperparathyroidism, hyperphosphatemia, decreased 1,25D, increased FGF-23, and decreased klotho expression [55]. A crosslink is reported between IL-6, a key mediator of inflammatory response, and the release of FGF-23. FGF-23 and the related α-Klotho modification as consequences of inflammation may be considered key mediators acting on the kidney and probably the critical link between the development of acute kidney damage and CKD and related CV morbidity [56].

The role of inflammatory response in sepsis-induced AKI was first recognized in viral infections such as cytomegalovirus, Epstein–Barr virus, and *influenzae* virus, as well as in bacterial infections, i.e., streptococcus infection and *Pneumocystis carinii jiroveci* [57]. In 90% of patients with sepsis-induced AKI, infections are due to either Gram-positive (mainly staphylococci and streptococci) or Gram-negative bacteria (predominantly *Escherichia coli*, *Pseudomonas aeruginosa*, and Klebsiella species). 

Sepsis-induced AKI is a clinical syndrome characterized by a systemic inflammatory response to an infective insult. However, infection may be proven or suspected, and the accompanying nonspecific systemic inflammatory response syndrome may also be triggered by noninfectious stimuli [58,59].

Among the possible risk factors of AKI, cytokine release syndrome has been described. Although previous studies focused on systemic hypotension and renal vasoconstriction as the primary pathophysiological mechanisms involved in sepsis-induced AKI, it has been now recognized that microvascular dysfunction and oxygen homeostasis impairment, leading to OS and hypoxemia, are principally involved. In sepsis-induced AKI, a central role is played by inflammatory mediators derived from pathogens, namely pathogen-associated molecular patterns (PAMPs), and activated immune cells, also known as damage-associated molecular patterns (DAMPs), that induce a release of proinflammatory cytokines such as TNF-α, IL-1α, and IL-6. These cytokines stimulate T cell activation in cooperation with tissue factors (Figure 2) [47]. 

Moreover, activation of the endothelium by circulating inflammatory cytokines leads to the increased expression of endothelial adhesion molecules, such as P-selectin, intracellular adhesion molecule-1 (ICAM-1), and vascular cell adhesion molecule-1 (VCAM-1) in the peritubular capillaries with leukocyte activation, which creates a vicious circle in the inflammatory response [47].

Recently, novel biomarkers of sepsis-induced AKI have been identified, including alpha 1-microglobulin (α1 m) [60]. More recently, other markers, including presepsin, a fragment of CD14; procalcitonin, currently recognized as suitable markers for the diagnosis of sepsis or severe sepsis [61]; neutrophil gelatinase-associated lipocalin (NGAL) [62]; soluble endothelial selectin (E-selectin); ICAM-1; VCAM-1; and urinary albumin-to-creatinine ratio (ACR), have particularly been considered in AKI diagnosis and prognosis [25,42].

Sepsis, acute pancreatitis, burns, and organ transplantation have similar widely described mechanisms that associate cytokines uncontrolled inflammatory response and AKI onset [43].

### 1.3. CKD, Inflammation, and SARS-CoV-2 

The role of inflammatory response has more recently been observed and described in the severe acute respiratory syndrome coronavirus 2 (SARS-CoV-2) infection that emerged from the novel coronavirus. Although SARS-CoV-2 disease (COVID) specifically affects respiratory apparatus with acute interstitial and alveolar pneumonia, other organs such as the kidney can be involved, as recently reported in the literature [63].

In SARS-CoV-2 infection, CKD stages 4 and 5 are recognized as the leading risk factors for hospitalization from COVID-19 and evolution to a severe form and RRT [64].

From the first description of SARS-CoV-2 infection in Wuhan, kidney involvement has been increasingly highlighted in numerous subsequent studies. More than 32% of COVID-19 patients have comorbidities, in particular CVD, DM, and AH, and in a high percentage of these patients, CKD is present. 

Angiotensin-converting enzyme 2 (ACE2) has been recognized as the first line of virus entry. Since 2002, interactions between the SARS-CoV spike protein and its host receptor, angiotensin-converting enzyme 2 (ACE2), were discovered and considered responsible for human-to-human virus transmission [65]. Strong evidence supports ACE2 as receptors for SARS-CoV-2, displayed on the principal target cells in the respiratory tract and lungs but also in the heart and kidney, in particular on glomerular and tubular cells [66].

In patients with SARS-CoV-2 infection, unlike in the Middle East respiratory syndrome coronavirus (MERS-CoV) or previous coronavirus infections, electron microscopic examination of postmortem kidney showed clusters of coronavirus particles with specific spikes in the tubular epithelium and podocytes. In these cases, diffuse proximal tubule injury with the loss of brush border, vacuolar degeneration, and necrosis was observed. SARS-CoV-2 was also detected in the cytoplasm of the renal proximal tubular epithelium as well as in the podocytes and distal tubules [67].

Erythrocyte aggregates were described as obstructing the lumen of renal capillaries without evidence of vasculitis, interstitial inflammation, or hemorrhage, highlighting evidence of the direct invasion of SARS-CoV-2 into kidney tissue. In addition, systemic effects caused by SARS-CoV-2 invasion such as systemic hypoxia, abnormal coagulation, rhabdomyolysis, or bacterial infections are often associated. All these factors contribute to AKI, acute on chronic kidney disease, and other clinical renal manifestations such as new-onset proteinuria or glycosuria associated with tubulointerstitial damage [67].

Through severe illness and hypoxia, many factors predispose patients to thrombotic events. Severe inflammatory response, critical illness, detection of anticardiolipin antibodies, and underlying traditional risk factors may all predispose patients to thrombotic events; moreover, therapies for treating COVID-19 may have adverse drug–drug interactions with antiplatelet agents and anticoagulants [68].

Kidney transplant recipients appear to be at particularly high risk for critical COVID-19 due to chronic immunosuppression and coexisting conditions such as CKD, AH, and DM. Kidney transplant recipients with COVID-19 present less fever as an initial symptom, lower CD3, CD4, and CD8 cell counts, and more rapid clinical progression than patients with COVID-19 in the general population. Very low CD3, CD4, and CD8 cell counts indirectly support the need to decrease doses of immunosuppressive agents in patients with COVID-19, especially in those who have received antithymocyte globulin, which decreases all T-cell subsets for many weeks [69,70].

The inflammatory response, as shown in COVID-19, recognizes the central role of “cytokine storm” as the excessive and uncontrolled release of proinflammatory cytokines associated with an uncontrolled inflammatory response. IL-2, IL-6, and TNF-α are the most important cytokines associated with cytokine storm and responsible for pathological alterations occurring, in particular, at the renal level [59].

### 1.4. CKD Inflammation, and Bone Disease

In recent years, bone has been revealed as a wide endocrine organ over the mere controller of calcium and phosphate equilibrium [71].

CKD is associated with the disruption of bone homeostasis that induces renal osteodystrophy, vascular and ectopic calcification, and the biochemical derangements of calcium-phosphate metabolism, identifying the syndrome called chronic kidney disease-mineral bone disorder (CKD-MBD) [71,72,73].

A central role is claimed for fibroblast growth factor 23 (FGF23) and Klotho. FGF23 is expressed by osteocytes and osteoblasts, mainly produced by osteocytes, and increases in the early stages of CKD, exerting an important regulatory effect in vitamin D and mineral metabolism [74,75]. Klotho, considered an antiaging protein, progressively reduces its levels along with the reduction of GFR. Disruption of FGF23 and Klotho levels is independently associated with an increased risk of early death in CKD patients, as demonstrated among incident HD patients with low vitamin D levels [76,77].

Proinflammatory mediators such as IL-1β, IL-6, and TNF-α and inflammatory markers such as CRP and fibrinogen are increased in CKD [13]. Higher FGF23 levels were shown to be independently associated with a higher concentration of proinflammatory mediators and inflammatory markers [14]. The link between FGF23 and inflammation is reciprocal and exerts a number of negative clinical effects. Inflammation directly stimulates osteocyte production by FGF23. Elevated levels of FGF23 induce the production of inflammatory mediators by activating FGF receptors 4 on hepatocytes in CKD [78]. The association between FGF23 and inflammation, together with the activation of FGF receptor 4 on cardiac myocytes that promote left ventricular hypertrophy and intramyocardial fibrosis responsible for cardiovascular events in CKD [79], represent an additional novel mechanism of mortality linked to CKD. 

Parathyroid hormone (PTH) may have a different action secondary to the CKD inflammatory state. Some evidence has shown that inflammation might cause irreversible oxidation into PTH methionine-sulfone residues. These changes in the three-dimensional structure modify the interaction between PTH and its receptor. The available commercial kits are not able to discriminate oxidized PTH. Therefore, in the inflammatory state, discrepancies may result in total PTH values and severity of bone and vascular pathology, resulting in a confounding factor in the interpretation of the PTH serum value and its pathophysiological action [80].

Lower levels of fetuin-A, a serum calcium-binding protein, are associated with an increased likelihood of extraskeletal calcification and high CPR levels in patients with CKD. Fetuin-A, CPR, and albumin modification are considered inflammatory biomarkers associated with increased mortality [37]. 

### 1.5. CKD, Inflammation, and Nutritional Status/Malnutrition 

Protein-energy wasting (PEW) and inflammation are the principal risk factors of malnutrition in HD patients. Malnutrition and inflammation often occur concomitantly in HD patients and correlate with elevated mortality reported in this population [81].

Causes of PEW in HD patients are numerous, recognizing inadequate nutrient-energy intake, nutrient losses during dialysis, hypercatabolism caused by comorbidities, and endocrine disorders or anemia, which can lead to inflammation. PEW can also be secondary to inflammation, as it is demonstrated that proinflammatory cytokines promote both catabolic processes and anorexia. Typically, HD patients with inflammation present conditions such as reduction in albumin synthesis and elevated CRP associated with hypocholesterolemia. The frequent association of inflammation and malnutrition in CKD characterizes the so-called “malnutrition–inflammation complex syndrome” (MICS), defined by a score with additional elements such as body mass index (BMI), serum albumin, and total iron-binding capacity [82]. Malnutrition, inflammation, and persistent infections determine the elevated morbidity and mortality observable in maintenance HD (MHD) patients, mostly as a consequence of CVDs. 

Moreover, in order to detect early-onset inflammation and malnutrition in HD patients before overt MICS onset, regular screening of prognostic inflammatory and nutritional index (PINI), calculated as alpha1-acid glycoprotein (a1-AG) CRP/albumin and transthyretin, has been suggested. The PINI score allows identifying very early patients at a higher risk of mortality and morbidity even in the absence of overt MICS, identifying patients with subclinical inflammation and/or malnutrition [20].

Inflammatory status associated with OS is a result of the imbalance between antioxidant defenses and free radical production. Hyperhomocysteinemia, OS, malnutrition, and uremic sarcopenia are highly prevalent in CKD, as demonstrated in stage 3/5 KDOQI, HD, peritoneal dialysis (PD) patients, and in kidney transplant patients. These conditions are associated with changes in early systemic indices of atherosclerosis and endothelial dysfunction, known as markers of worse prognosis, including bioimpedance analysis (BIA), hand-grip strength (HGS), intima-media thickness (IMT), flow-mediated dilation (FMD), and epicardial adipose tissue (EAT). Several mechanisms are considered that can affect muscle mass, such as vitamin D deficiency, HDL levels, low protein intake, physical inactivity, metabolic acidosis, and inflammation. 

In CKD sarcopenic patients, modifications in cognitive function and mood, evaluated with the mini-mental state examination (MMSE) and geriatric depression scale (GDS), have been recognized [83,84]. 

### 1.6. CKD Inflammatory Status, and Treatment

Treatment of CKD recommends the control of traditional risk factors such as AH, hyperglycemia, and dyslipidemia with common pharmacological tools, i.e., angiotensin-converting-enzyme (ACE) inhibitors or angiotensin-receptor blocker (ARB) and statins. Otherwise, metabolic diseases such as nephropathy, hypertension, and diabetes are associated with increased expression of mineralocorticoid receptors (MRs) and enhanced aldosterone signaling. Aldosterone, through stimulation of MR, exerts a series of effects on renal tissue through the release of proinflammatory factors, synthesis of profibrotic cytokines, and induction of oxidative stress [85]. 

This activation leads to the development of microangiopathy in renal blood vessels, glomerulosclerosis, an increase in protein excretion, and the deterioration of GFR with negative effects on both myocardial and renal district [86,87]. 

Mineralocorticoid receptor antagonists (MRAs) have demonstrated beneficial effects on renal inflammation in various animal studies, directly regulating inflammatory cell function and indirectly suppressing proinflammatory cytokines, chemoattractants, and pro-oxidants, increasing anti-inflammatory cytokines in kidneys [88]. Spironolactone and eplerenone in particular demonstrated beneficial effects, reducing the kidney’s inflammatory markers [89,90].

Hyperkalemia as a first side effect, and undesirable effects such as gynecomastia, dysmenorrhea, and impotence associated with steroidal MRA, have frequently caused discontinuation of treatment. The new generation of nonsteroidal MRAs are less likely to cause hyperkalemia at a therapeutic dosage and may be an additional tool in the treatment of complications related to renal and metabolic diseases. Finer none recently demonstrated lowering risks of CKD progression and CV events in patients with type 2 diabetes mellitus (T2DM), with no significantly increased risk of discontinuation of therapy because of hyperkalemia [91].

Improved clinical outcomes in T2DM, in terms of a reduction in CV events and renal outcomes such as albuminuria and eGFR decline, are reported with the administration of sodium–glucose cotransporter type 2 (SGLT2) inhibitors [92,93].

Sodium–glucose cotransporter 2 (SGLT-2) inhibitors are hypothesized to exert a positive effect on inflammatory markers in T2DM patients with few data from small pilot studies, which confirms a reduction in leptin levels, C-reactive protein (CRP), TNF-α, IL-6, and interferon-gamma (IFN-γ) [94,95]. In particular, animal models demonstrated the mechanism of action of SGLT2 inhibitors in reducing inflammation in diabetic kidneys, reporting that empagliflozin and dapagliflozin brought reductions in OS and markers of inflammation and fibrosis, attenuate histological evidence of nephropathy, prevent the enhanced expression of inflammatory genes, and reduce IL-6 urinary concentrations [96]. Long-term treatment with the SGLT2 inhibitor dapagliflozin ameliorates glucose homeostasis and diabetic nephropathy in db/db mice and slows the progression of renal complications through the suppression of renal inflammation, ER stress, and apoptosis in prediabetic rats [97,98]. 

The increase in the inflammatory status associated with OS may be considered a target for clinical management, as shown by the effect of reduction of inflammatory biomarkers observed after oral food supplementation anti-inflammatory action treatment [99]. The chronic treatment with statins seems to enhance antioxidant defense systems of hemodialysis patients increasing the availability of selenium [25].

In uremic patients with OS, it is common to observe a vitamin C deficiency partially due to a reduced intake [100,101]. Therefore, several studies have experimented with oral food supplements with high ascorbic acid added with natural bioactive compounds such as Polyphenols, in chronic degenerative noncommunicable diseases and CKD, or a daily supplement of α-lipoic acid (ALA) in patients with autosomal dominant polycystic kidney disease (ADPKD) treated with α-lipoic acid (ALA) with the aim to counteract the abnormal increase in OS and inflammatory status. In these cases, a significant improvement of metabolic, inflammatory, and endothelial dysfunction indexes was reached, with a significant improvement in NADPH oxidase 2 (NOX2), as confirmed by the monitoring in these cohorts patients of CRP, erythrocyte sedimentation rate, early erythrocyte glutathione-transferase, and human oxidized serum albumin [102,103]. 

As demonstrated in experimental studies, it is plausible that either HIF-1 alpha deficiency or activation may influence kidney injury and HIF-2 alpha expression modification that ameliorates renal injury by ischemia, as observed in HIF-2-alpha-deficient mice [104].

### 1.7. Personal Experience

Our research group explored CKD and inflammation in different settings, evaluating the association between inflammation and renal dysfunction and defining the several mechanisms involved in their complexity.

In particular, we explored the onset of CKD de novo after AKI in different degrees of hypoxic-ischemic damage and in sepsis after organ transplantation, recognizing a role for ischemia-reperfusion injury in the development of AKI and CKD [47,105,106].

Sarcopenic patients with CKD stages 3/5 and hemodialysis, peritoneal dialysis, and postkidney transplant showed hypercatabolism leading to inflammation, higher values of intima-media thickness, and epicardial adipose tissue with total cholesterol and high-density lipoprotein cholesterol with respect to nonsarcopenic patients [83].

In another cross-sectional, observational study of our research group, 226 patients diagnosed with cystic fibrosis (CF), we reported a significant association between lower eGFR value and alteration of metabolic CV index, with a significant negative correlation between eGFR, triglycerides, and serum uric acid, which is involved in endothelial dysfunction by increasing inflammation and oxidative stress. Moreover, an association between significantly a lower estimated eGFR and lung transplantation was also reported [107].

On the other hand, studies of our group in a nutritional setting evaluated the action of α-lipoic acid (ALA) daily supplementation in patients with autosomal dominant polycystic kidney disease (ADPKD), demonstrating a significant improvement of metabolic, inflammatory, endothelial dysfunction, and OS indexes, namely the amelioration of serum glucose and uric acid, CRP, renal resistive index, and NADPH oxidase 2 (NOX2), with the aim to counteract the abnormal increase of OS and inflammatory status [103].

The administration of bioactive compounds (oral food supplementation (OFS) rich in citrus fruit and rosehip extracts) in nephropathic patients was evaluated and was shown to be associated with a significant reduction of laboratory parameters related to inflammation such as CRP, erythrocyte sedimentation rate, and platelet-to-lymphocyte ratio [102].

Moreover, other natural bioactive compounds (OFS based on *Capsicum annuum* L., *Garcinia cambogia*, *Centella asiatica* L., artichoke, and *Aesculus hippocastanum* L.) induce an improvement in lipid metabolism, inflammatory state, and body composition in a group of nephropathic male patients [99] and ultramicronized palmitoylethanolamide (um-PEA) considered an potential adjuvant treatment in COVID-19 patients [12].

The minor polar compounds (MPCs) present in extra virgin olive oil (EVOO) exert an anti-inflammatory and antioxidant effect [108]. The use of EVOO with high-content MPCs demonstrated an improvement of the inflammatory state of the OS of the body composition and of the renal function in nephropathic patients, with long-term beneficial effects, probably related to the positive changes induced by polyphenols on the gut microbiota [109]. 

The multidisciplinary nature of our research on CKD highlights the complexity of mechanisms involved in linking CKD to systemic manifestations.

## 2. Conclusions

CKD is classically defined as the gradual loss of kidney function or the abnormality of kidney structure with implications for health, characterized by the presence of markers of kidney damage and a decrease in the glomerular filtration rate [110].

With our revision, we explored CKD from a different point of view, describing CKD as a systemic inflammatory syndrome characterized by complex dynamic mechanisms developing from multiple target organs and leading to tissue injury and contributing to increased morbidity and mortality.

We highlighted the role of inflammation as a linking condition present in all clinical, biological, and biochemical aspects of CKD, from traditional risk factors of renal disease, including AH, DM, and dyslipidemia, to cellular and immunologic responses involving multiple proinflammatory processes.

On the basis of these elements, the traditional view of CKD as a disease characterized by the progressive loss of specific functions involving individual organs and systems evolves toward a dynamic relationship between organs mediated by complex inflammatory mechanisms, actively contributing to the expression of comorbidities and the progression of CKD.

Classical features associated with CKD, such as mineral bone disease, CVD, AH, DM, obesity, dysfunctional adipose tissue and insulin resistance, malnutrition, infection, hypoxia, and AKI, develop due to the syndromes associated with CKD and become active contributors to CKD onset and evolution through MBD-CKD syndrome, MICS, PEW, and uremic sarcopenia, recognizing the central role of a low-grade inflammatory status and cytokine release.

In accordance with this view and our personal experience, the depth comprehension of the mechanisms underlying CKD as a systemic disease may open novel possibilities for specific treatments in addition to the replacement of individual organ loss of function, acting on common mechanisms underlying the disease, to improve the management of disease evolution and correlated morbidities and reduce mortality related to CKD.

## Data Availability

Not applicable.

## References

[1] Lopes J.A., Raimundo M. (2011). Metabolic syndrome, chronic kidney disease, and cardiovascular disease: A dynamic and life-threatening triad. Cardiol. Res. Pract..

[2] Bikbov B., Purcell C.A., Levey A.S., Smith M., Abdoli A., Abebe M., Adebayo O.M., Afarideh M., Agarwal S.K., Agudelo-Botero M. (2020). Global, regional, and national burden of chronic kidney disease, 1990–2017: A systematic analysis for the Global Burden of Disease Study 2017. Lancet.

[3] Liyanage T., Ninomiya T., Jha V., Neal B., Patrice H.M., Okpechi I., Zhao M.H., Lv J., Garg A.X., Knight J. (2015). Worldwide access to treatment for end-stage kidney disease: A systematic review. Lancet.

[4] Kazancioǧlu R. (2013). Risk factors for chronic kidney disease: An update. Kidney Int. Suppl..

[5] Hsu R.K., Hsu C.Y. (2016). The Role of Acute Kidney Injury in Chronic Kidney Disease. Semin. Nephrol..

[6] Liakopoulos V., Roumeliotis S., Gorny X., Dounousi E., Mertens P.R. (2017). Oxidative Stress in Hemodialysis Patients: A Review of the Literature. Oxid. Med. Cell. Longev..

[7] Levey A.S., Coresh J. (2012). Chronic kidney disease. Lancet.

[8] Podkowińska A., Formanowicz D. (2020). Chronic Kidney Disease as Oxidative Stress- and Inflammatory-Mediated Cardiovascular Disease. Antioxidants.

[9] Rossaint J., Oehmichen J., Van Aken H., Reuter S., Pavenstädt H.J., Meersch M., Unruh M., Zarbock A. (2016). FGF23 signaling impairs neutrophil recruitment and host defense during CKD. J. Clin. Invest..

[10] Rosengren B.I., Sagstad S.J., Karlsen T.V., Wiig H. (2013). Isolation of interstitial fluid and demonstration of local proinflammatory cytokine production and increased absorptive gradient in chronic peritoneal dialysis. Am. J. Physiol. - Ren. Physiol..

[11] Mihai S., Codrici E., Popescu I.D., Enciu A.M., Albulescu L., Necula L.G., Mambet C., Anton G., Tanase C. (2018). Inflammation-related mechanisms in chronic kidney disease prediction, progression, and outcome. J. Immunol. Res..

[12] Noce A., Albanese M., Marrone G., Di Lauro M., Pietroboni Zaitseva A., Palazzetti D., Guerriero C., Paolino A., Pizzenti G., Di Daniele F. (2021). Ultramicronized Palmitoylethanolamide (um-PEA): A New Possible Adjuvant Treatment in COVID-19 patients. Pharmaceuticals.

[13] Gupta J., Mitra N., Kanetsky P.A., Devaney J., Wing M.R., Reilly M., Shah V.O., Balakrishnan V.S., Guzman N.J., Girndt M. (2012). Association between albuminuria, kidney function, and inflammatory biomarker profile in CKD in CRIC. Clin. J. Am. Soc. Nephrol..

[14] Munoz Mendoza J., Isakova T., Cai X., Bayes L.Y., Faul C., Scialla J.J., Lash J.P., Chen J., He J., Navaneethan S. (2017). Inflammation and elevated levels of fibroblast growth factor 23 are independent risk factors for death in chronic kidney disease. Kidney Int..

[15] Honda H., Qureshi A.R., Heimbürger O., Barany P., Wang K., Pecoits-Filho R., Stenvinkel P., Lindholm B. (2006). Serum albumin, C-reactive protein, interleukin 6, and fetuin a as predictors of malnutrition, cardiovascular disease, and mortality in patients with ESRD. Am. J. Kidney Dis..

[16] Kim H.J., Vaziri N.D. (2010). Contribution of impaired Nrf2-Keap1 pathway to oxidative stress and inflammation in chronic renal failure. Am. J. Physiol. - Ren. Physiol..

[17] Ori Y., Bergman M., Bessler H., Zingerman B., Levy-Drummer R.S., Gafter U., Salman H. (2013). Cytokine secretion and markers of inflammation in relation to acidosis among chronic hemodialysis patients. Blood Purif..

[18] Motohashi H., Yamamoto M. (2004). Nrf2-Keap1 defines a physiologically important stress response mechanism. Trends Mol. Med..

[19] Jaramillo M.C., Zhang D.D. (2013). The emerging role of the Nrf2-Keap1 signaling pathway in cancer. Genes Dev..

[20] Dessì M., Noce A., Agnoli A., De Angelis S., Fuiano L., Tozzo C., Taccone-Gallucci M., Fuiano G., Federici G. (2009). The usefulness of the prognostic inflammatory and nutritional index (PINI) in a haemodialysis population. Nutr. Metab. Cardiovasc. Dis..

[21] Noce A., Canale M.P., Capria A., Rovella V., Tesauro M., Splendiani G., Annicchiarico-Petruzzelli M., Manzuoli M., Simonetti G., Di Daniele N. (2015). Coronary artery calcifications predict long term cardiovascular events in non diabetic Caucasian hemodialysis patients. Aging.

[22] Morrone L.F., Bolasco P., Camerini C., Cianciolo G., Cupisti A., Galassi A., Mazzaferro S., Russo D., Russo L., Cozzolino M. (2016). Vitamin D in patients with chronic kidney disease: A position statement of the Working Group “Trace Elements and Mineral Metabolism” of the Italian Society of Nephrology. J. Nephrol..

[23] Pastore A., Noce A., Di Giovamberardino G., De Stefano A., Callà C., Zenobi R., Dessì M., Di Daniele N. (2015). Homocysteine, cysteine, folate and vitamin B12 status in type 2 diabetic patients with chronic kidney disease. J. Nephrol..

[24] Noce A., Marrone G., Rovella V., Cusumano A., Di Daniele N., Casasco M. (2018). Beneficial effects of physical activity on uremic sarcopenia. Med. dello Sport.

[25] Taccone-Gallucci M., Noce A., Bertucci P., Fabbri C., Manca-di-Villahermosa S., Della-Rovere F.R., De Francesco M., Lonzi M., Federici G., Scaccia F. (2010). Chronic treatment with statins increases the availability of selenium in the antioxidant defence systems of hemodialysis patients. J. Trace Elem. Med. Biol..

[26] Ravarotto V., Simioni F., Pagnin E., Davis P.A., Calò L.A. (2018). Oxidative stress – chronic kidney disease – cardiovascular disease: A vicious circle. Life Sci..

[27] Anders H.J., Andersen K., Stecher B. (2013). The intestinal microbiota, a leaky gut, and abnormal immunity in kidney disease. Kidney Int..

[28] Kshirsagar A.V., Craig R.G., Moss K.L., Beck J.D., Offenbacher S., Kotanko P., Klemmer P.J., Yoshino M., Levin N.W., Yip J.K. (2009). Periodontal disease adversely affects the survival of patients with end-stage renal disease. Kidney Int..

[29] Icardi A., Paoletti E., De Nicola L., Mazzaferro S., Russo R., Cozzolino M. (2013). Renal anaemia and EPO hyporesponsiveness associated with vitamin D deficiency: The potential role of inflammation. Nephrol. Dial. Transplant..

[30] Autier P., Boniol M., Pizot C., Mullie P. (2014). Vitamin D status and ill health: A systematic review. Lancet Diabetes Endocrinol..

[31] Murai I.H., Fernandes A.L., Sales L.P., Pinto A.J., Goessler K.F., Duran C.S.C., Silva C.B.R., Franco A.S., Macedo M.B., Dalmolin H.H.H. (2021). Effect of a Single High Dose of Vitamin D3 on Hospital Length of Stay in Patients With Moderate to Severe COVID-19: A Randomized Clinical Trial. Jama.

[32] Chintam K., Chang A.R. (2021). Strategies to Treat Obesity in Patients With CKD. Am. J. Kidney Dis..

[33] Iglesias P., Díez J.J. (2010). Adipose tissue in renal disease: Clinical significance and prognostic implications. Nephrol. Dial. Transplant..

[34] Targher G., Chonchol M.B., Byrne C.D. (2014). CKD and nonalcoholic fatty liver disease. Am. J. kidney Dis..

[35] Marcuccilli M., Chonchol M. (2016). NAFLD and chronic kidney disease. Int. J. Mol. Sci..

[36] Satapathy S.K., Garg S., Chauhan R., Sakhuja P., Malhotra V., Sharma B.C., Sarin S.K. (2004). Beneficial effects of tumor necrosis factor-α inhibition by pentoxifylline on clinical, biochemical, and metabolic parameters of patients with nonalcoholic steatohepatitis. Am. J. Gastroenterol..

[37] Laclair R., O’Neal K., Ofner S., Sosa M.J., Labarrere C.A., Moe S.M. (2008). Precision of biomarkers to define chronic inflammation in CKD. Am. J. Nephrol..

[38] Yeun J.Y., Levine R.A., Mantadilok V., Kaysen G.A. (2020). C-reactive protein predicts all-cause and cardiovascular mortality in hemodialysis patients. Am. J. Kidney Dis..

[39] Stenvinkel P., Barany P., Heimbürger O., Pecoits-Filho R., Lindholm B. (2002). Mortality, malnutrition, and atherosclerosis in ESRD: What is the role of interleukin-6?. Kidney Int. Suppl..

[40] Ketteler M., Bongartz P., Westenfeld R., Wildberger J.E., Mahnken A.H., Böhm R., Metzger T., Wanner C., Jahnen-Dechent W., Floege J. (2003). Association of low fetuin-A (AHSG) concentrations in serum with cardiovascular mortality in patients on dialysis: A cross-sectional study. Lancet.

[41] Belayev L.Y., Palevsky P.M. (2014). The link between acute kidney injury and chronic kidney disease. Curr. Opin. Nephrol. Hypertens..

[42] Chawla L.S., Eggers P.W., Star R.A., Kimmel P.L. (2014). Acute Kidney Injury and Chronic Kidney Disease as Interconnected Syndromes. N. Engl. J. Med..

[43] Wald R., Quinn R.R., Luo J., Li P., Scales D.C., Mamdani M.M., Ray J.G. (2009). Chronic dialysis and death among survivors of acute kidney injury requiring dialysis. JAMA.

[44] (2012). Kidney Disease: Improving Global Outcomes (KDIGO) Acute Kidney Injury Work Group KDIGO Clinical Practice Guideline for Acute Kidney Injury. Kidney Int. Suppl..

[45] Chawla L.S., Amdur R.L., Amodeo S., Kimmel P.L., Palant C.E. (2011). The severity of acute kidney injury predicts progression to chronic kidney disease. Kidney Int..

[46] McMahon G.M. (2013). Biomarkers in Nephrology. Am. J. kidney Dis..

[47] Umbro I., Gentile G., Tinti F., Muiesan P., Mitterhofer A.P. (2016). Recent advances in pathophysiology and biomarkers of sepsis-induced acute kidney injury. J. Infect..

[48] Quinto B.M.R., Iizuka I.J., Monte J.C.M., Santos B.F., Pereira V., Durão M.S., Dalboni M.A., Cendoroglo M., Santos O.F.P., Batista M.C. (2015). TNF-α depuration is a predictor of mortality in critically ill patients under continuous veno-venous hemodiafiltration treatment. Cytokine.

[49] Malard B., Lambert C., Kellum J.A. (2019). “In vitro comparison of the adsorption of inflammatory mediators by blood purification devices”: A misleading article for clinical practice?. Intensive Care Med. Exp..

[50] Semenza G.L. (2001). HIF-1, O2, and the 3 PHDs: How animal cells signal hypoxia to the nucleus. Cell.

[51] Gunaratnam L., Bonventre J.V. (2009). HIF in kidney disease and development. J. Am. Soc. Nephrol..

[52] Maxwell P. (2003). HIF-1: An Oxygen Response System with Special Relevance to the Kidney. J. Am. Soc. Nephrol..

[53] Haase V.H. (2006). The VHL/HIF oxygen-sensing pathway and its relevance to kidney disease. Kidney Int..

[54] Norman J.T., Clark I.M., Garcia P.L. (2000). Hypoxia promotes fibrogenesis in human renal fibroblasts. Kidney Int..

[55] Leaf D.E., Christov M. (2019). Dysregulated Mineral Metabolism in AKI. Semin. Nephrol..

[56] Gao L., Zhong X., Jin J., Li J., Meng X.M. (2020). Potential targeted therapy and diagnosis based on novel insight into growth factors, receptors, and downstream effectors in acute kidney injury and acute kidney injury-chronic kidney disease progression. Signal Transduct. Target. Ther..

[57] Bozzette S.A., Sattler F.R., Chiu J., Wu A.W., Gluckstein D., Kemper C., Bartok A., Niosi J., Abramson I., Coffman J. (1990). A controlled trial of early adjunctive treatment with corticosteroids for Pneumocystis carinii pneumonia in the acquired immunodeficiency syndrome. California Collaborative Treatment Group. New English J. Med..

[58] Cunha F.Q., Assreuy J., Moss D.W., Rees D., Leal L.M., Moncada S., Carrier M., O’Donnell C.A., Liew F.Y. (1994). Differential induction of nitric oxide synthase in various organs of the mouse during endotoxaemia: Role of TNF-alpha and IL-1-beta. N. Engl. J. Med..

[59] Tisoncik J.R., Korth M.J., Simmons C.P., Farrar J., Martin T.R., Katze M.G. (2012). Into the Eye of the Cytokine Storm. Microbiol. Mol. Biol. Rev..

[60] Terzi I., Papaioannou V., Papanas N., Dragoumanis C., Petala A., Theodorou V., Gioka T., Vargemezis V., Maltezos E., Pneumatikos I. (2014). Alpha1-microglobulin as an early biomarker of sepsis-associated acute kidney injury: A prospective cohort study. Hippokratia.

[61] Cho S.Y., Choi J.H. (2014). Biomarkers of Sepsis. Infect. Chemother..

[62] Haase M., Bellomo R., Devarajan P., Schlattmann P., Haase-Fielitz A., Bagshaw S.M., Bogle R., Changchun C., Constantin J.M., Cruz D. (2009). Accuracy of Neutrophil Gelatinase-Associated Lipocalin (NGAL) in Diagnosis and Prognosis in Acute Kidney Injury: A Systematic Review and Meta-analysis. Am. J. Kidney Dis..

[63] Xu J., Zhao S., Teng T., Abdalla A.E., Zhu W., Xie L., Wang Y., Guo X. (2020). Systematic Comparison of Two Animal-to-Human Transmitted Human Coronaviruses: SARS-CoV-2 and SARS-CoV. Viruses.

[64] Cheng Y., Luo R., Wang K., Zhang M., Wang Z., Dong L., Li J., Yao Y., Ge S., Xu G. (2020). Kidney impairment is associated with in-hospital death of COVID-19 patients. medRxiv.

[65] Lee N., Hui D., Wu A., Chan P., Cameron P., Joynt G., Ahuja A.T., van der Gast C. (2003). A Major Outbreak of Severe Acute Respiratory Syndrome in Hong Kong. N. Engl. J. Med..

[66] Wan Y., Shang J., Graham R., Baric R.S., Li F. (2020). Receptor Recognition by the Novel Coronavirus from Wuhan: An Analysis Based on Decade-Long Structural Studies of SARS Coronavirus. J. Virol..

[67] Hua S., Ming Y., Cheng W., Li-Xia Y., Fang T., Zhu H.-Y. (2020). Renal histopathological analysis of 26 postmortem findings of patients with COVID-19 in China. Kidney Int..

[68] Yan Z., Meng X., Shulan Z., Peng X., Wei C., Wei J., Huan C., Xin D., Hua Z., Hongmin Z. (2020). Coagulopathy and Antiphospholipid Antibodies in Patients with Covid-19. N. Engl. J. Med..

[69] Akalin E., Azzi Y., Bartash R., Seethamraju H., Parides M., Hemmige V., Ross M. (2020). Covid-19 and Kidney Transplantation. N. Engl. J. Med..

[70] Naicker S., Yang C.W., Hwang S.J., Liu B.C., Chen J.H., Jha V. (2020). The Novel Coronavirus 2019 epidemic and kidneys. Kidney Int..

[71] Cozzolino M., Ureña-Torres P., Vervloet M.G., Brandenburg V., Bover J., Goldsmith D., Larsson T.E., Massy Z.A., Mazzaferro S. (2014). Is chronic kidney disease-mineral bone disorder (CKD-MBD) really a syndrome?. Nephrol. Dial. Transplant..

[72] Mazzaferro S., Bagordo D., De Martini N., Pasquali M., Rotondi S., Tartaglione L., Stenvinkel P. (2021). Inflammation, Oxidative Stress, and Bone in Chronic Kidney Disease in the Osteoimmunology Era. Calcif. Tissue Int..

[73] Moe S., Drüeke T., Cunningham J., Goodman W., Martin K., Olgaard K., Ott S., Sprague S., Lameire N., Eknoyan G. (2006). Definition, evaluation, and classification of renal osteodystrophy: A position statement from Kidney Disease: Improving Global Outcomes (KDIGO). Kidney Int..

[74] Cozzolino M., Mazzaferro S. (2010). The fibroblast growth factor 23: A new player in the field of cardiovascular, bone and renal disease. Curr. Vasc. Pharmacol..

[75] Grabner A., Mazzaferro S., Cianciolo G., Krick S., Capelli I., Rotondi S., Ronco C., La Manna G., Faul C. (2017). Fibroblast Growth Factor 23: Mineral Metabolism and beyond. Contrib. Nephrol..

[76] Isakova T., Xie H., Yang W., Xie D., Anderson A.H., Scialla J., Wahl P., Gutiérrez O.M., Steigerwalt S., He J. (2011). Fibroblast growth factor 23 and risks of mortality and end-stage renal disease in patients with chronic kidney disease. JAMA J. Am. Med. Assoc..

[77] Wolf M., Shah A., Gutierrez O., Ankers E., Monroy M., Tamez H., Steele D., Chang Y., Camargo C.A., Tonelli M. (2007). Vitamin D levels and early mortality among incident hemodialysis patients. Kidney Int..

[78] Singh S., Grabner A., Yanucil C., Schramm K., Czaya B., Krick S., Czaja M.J., Bartz R., Abraham R., Di Marco G.S. (2016). Fibroblast growth factor 23 directly targets hepatocytes to promote inflammation in chronic kidney disease. Kidney Int..

[79] Scialla J.J., Xie H., Rahman M., Anderson A.H., Isakova T., Ojo A., Zhang X., Nessel L., Hamano T., Grunwald J.E. (2014). Fibroblast growth factor-23 and cardiovascular events in CKD. J. Am. Soc. Nephrol..

[80] Mazzaferro S., Tartaglione L., Rotondi S., Bover J., Goldsmith D., Pasquali M. (2014). News on Biomarkers in CKD-MBD. Semin. Nephrol..

[81] Kalantar-Zadeh K., Kopple J.D. (2001). Relative contributions of nutrition and inflammation to clinical outcome in dialysis patients. Am. J. Kidney Dis..

[82] Enia G., Sicuso C., Alati G., Zoccali C., Pustorino D., Biondo A. (1993). Subjective global assessment of nutrition in dialysis patients. Nephrol. Dial. Transplant..

[83] Lai S., Muscaritoli M., Andreozzi P., Sgreccia A., De Leo S., Mazzaferro S., Mitterhofer A.P., Pasquali M., Protopapa P., Spagnoli A. (2019). Sarcopenia and cardiovascular risk indices in patients with chronic kidney disease on conservative and replacement therapy. Nutrition.

[84] Moorthi R.N., Avin K.G. (2017). Clinical relevance of sarcopenia in chronic kidney disease. Curr. Opin. Nephrol. Hypertens..

[85] Bauersachs J., Jaisser F., Toto R. (2015). Mineralocorticoid receptor activation and mineralocorticoid receptor antagonist treatment in cardiac and renal diseases. Hypertension.

[86] Rocha R., Stier C.T., Kifor I., Ochoa-Maya M.R., Rennke H.G., Williams G.H., Adler G.K. (2000). Aldosterone: A mediator of myocardial necrosis and renal arteriopathy. Endocrinology.

[87] Quinkler M., Zehnder D., Eardley K.S., Lepenies J., Howie A.J., Hughes S.V., Cockwell P., Hewison M., Stewart P.M. (2005). Increased expression of mineralocorticoid effector mechanisms in kidney biopsies of patients with heavy proteinuria. Circulation.

[88] Patel V., Joharapurkar A., Jain M. (2020). Role of mineralocorticoid receptor antagonists in kidney diseases. Drug Dev. Res..

[89] Orena S., Maurer T.S., She L., Eudy R., Bernardo V., Dash D., Loria P., Banker M.E., Tugnait M., Okerberg C.V. (2013). PF-03882845, a non-steroidal mineralocorticoid receptor antagonist, prevents renal injury with reduced risk of hyperkalemia in an animal model of nephropathy. Front. Pharmacol..

[90] Zitt E., Eller K., Huber J.M., Kirsch A.H., Tagwerker A., Mayer G., Rosenkranz A.R. (2011). The selective mineralocorticoid receptor antagonist eplerenone is protective in mild anti-GBM glomerulonephritis. Int. J. Clin. Exp. Pathol..

[91] Bakris G.L., Agarwal R., Anker S.D., Pitt B., Ruilope L.M., Rossing P., Kolkhof P., Nowack C., Schloemer P., Joseph A. (2020). Effect of Finerenone on Chronic Kidney Disease Outcomes in Type 2 Diabetes. N. Engl. J. Med..

[92] Zinman B., Wanner C., Lachin J.M., Fitchett D., Bluhmki E., Hantel S., Mattheus M., Devins T., Johansen O.E., Woerle H.J. (2015). Empagliflozin, cardiovascular outcomes, and mortality in type 2 diabetes. N. Engl. J. Med..

[93] Perkovic V., de Zeeuw D., Mahaffey K.W., Fulcher G., Erondu N., Shaw W., Barrett T.D., Weidner-Wells M., Deng H., Matthews D.R. (2018). Canagliflozin and renal outcomes in type 2 diabetes: Results from the CANVAS Program randomised clinical trials. Lancet Diabetes Endocrinol..

[94] Garvey W.T., Van Gaal L., Leiter L.A., Vijapurkar U., List J., Cuddihy R., Ren J., Davies M.J. (2018). Effects of canagliflozin versus glimepiride on adipokines and inflammatory biomarkers in type 2 diabetes. Metabolism..

[95] Tan S.A., Tan L. (2018). Empagliflozin and Canagliflozin Attenuate Inflammatory Cytokines Interferon-Λ, Tumor Necrosis Factor-Α, Interleukin-6: Possible Mechanism of Decreasing Cardiovascular Risk in Diabetes Mellitus. J. Am. Coll. Cardiol..

[96] Kawanami D., Matoba K., Takeda Y., Nagai Y., Akamine T., Yokota T., Sango K., Utsunomiya K. (2017). SGLT2 inhibitors as a therapeutic option for diabetic nephropathy. Int. J. Mol. Sci..

[97] Terami N., Ogawa D., Tachibana H., Hatanaka T., Wada J., Nakatsuka A., Eguchi J., Sato Horiguchi C., Nishii N., Yamada H. (2014). Long-term treatment with the sodium glucose cotransporter 2 inhibitor, dapagliflozin, ameliorates glucose homeostasis and diabetic nephropathy in db/db mice. PLoS One.

[98] Dekkers C.C.J., Petrykiv S., Laverman G.D., Cherney D.Z., Gansevoort R.T., Heerspink H.J.L. (2018). Effects of the SGLT-2 inhibitor dapagliflozin on glomerular and tubular injury markers. Diabetes Obes. Metab..

[99] Noce A., Marrone G., Di Lauro M., Urciuoli S., Pietroboni Zaitseva A., Wilson Jones G., Di Daniele N., Romani A. (2020). Cardiovascular Protection of Nephropathic Male Patients by Oral Food Supplements. Cardiovasc. Ther..

[100] Raimann J.G., Levin N.W., Craig R.G., Sirover W., Kotanko P., Handelman G. (2013). Is Vitamin C Intake too Low in Dialysis Patients?. Semin. Dial..

[101] Gillis K., Stevens K.K., Bell E., Patel R.K., Jardine A.G., Morris S.T.W., Schneider M.P., Delles C., Mark P.B. (2018). Ascorbic acid lowers central blood pressure and asymmetric dimethylarginine in chronic kidney disease. Clin. Kidney J..

[102] Noce A., Bocedi A., Campo M., Marrone G., Di Lauro M., Cattani G., Di Daniele N., Romani A. (2020). A pilot study of a natural food supplement as new possible therapeutic approach in chronic kidney disease patients. Pharmaceuticals.

[103] Lai S., Petramala L., Muscaritoli M., Cianci R., Mazzaferro S., Mitterhofer A.P., Pasquali M., D’Ambrosio V., Carta M., Ansuini M. (2020). Α-Lipoic Acid in Patients With Autosomal Dominant Polycystic Kidney Disease. Nutrition.

[104] Kojima I., Tanaka T., Inagi R., Kato H., Yamashita T., Sakiyama A., Ohneda O., Takeda N., Sata M., Miyata T. (2007). Protective role of hypoxia-inducible factor-2α against ischemic damage and oxidative stress in the kidney. J. Am. Soc. Nephrol..

[105] Kalisvaart M., Schlegel A., Umbro I., De Haan J.E., Scalera I., Polak W.G., Ijzermans J.N.M., Mirza D.F., Perera M.T.P.R., Isaac J.I. (2018). The Impact of Combined Warm Ischemia Time on Development of Acute Kidney Injury in Donation after Circulatory Death Liver Transplantation: Stay Within the Golden Hour. Transplantation.

[106] Umbro I., Tinti F., Scalera I., Evison F., Gunson B., Sharif A., Ferguson J., Muiesan P., Mitterhofer A.P. (2016). Acute kidney injury and post-reperfusion syndrome in liver transplantation. World J. Gastroenterol..

[107] Lai S., Mazzaferro S., Mitterhofer A.P., Bonci E., Marotta P.G., Pelligra F., Murciano M., Celani C., Troiani P., Cimino G. (2019). Renal involvement and metabolic alterations in adults patients affected by cystic fibrosis. J. Transl. Med..

[108] Romani A., Bernini R., Noce A., Urciuoli S., Lauro M.D., Zaitseva A.P., Marrone G., Daniele N. (2020). Di Potential beneficial effects of extra virgin olive oils characterized by high content in minor polar compounds in nephropathic patients: A pilot study. Molecules.

[109] Noce A., Marrone G., Urciuoli S., Di Daniele F., Di Lauro M., Zaitseva A.P., Di Daniele N., Romani A. (2021). Usefulness of extra virgin olive oil minor polar compounds in the management of chronic kidney disease patients. Nutrients.

[110] Kidney Disease: Improving Global Outcomes (KDIGO) CKD Work Group (2013). KDIGO 2012 Clinical Practice Guideline for the Evaluation and Management of Chronic Kidney Disease. Kidney Int..

